# Interactions between genes involved in physiological dysregulation and axon guidance: role in Alzheimer’s disease

**DOI:** 10.3389/fgene.2023.1236509

**Published:** 2023-08-31

**Authors:** Konstantin G. Arbeev, Svetlana Ukraintseva, Olivia Bagley, Hongzhe Duan, Deqing Wu, Igor Akushevich, Eric Stallard, Alexander Kulminski, Kaare Christensen, Mary F. Feitosa, Jeffrey R. O’Connell, Daniel Parker, Heather Whitson, Anatoliy I. Yashin

**Affiliations:** ^1^ Biodemography of Aging Research Unit, Social Science Research Institute, Duke University, Durham, NC, United States; ^2^ Danish Aging Research Center, Department of Public Health, University of Southern Denmark, Odense, Denmark; ^3^ Division of Statistical Genomics, Department of Genetics, Washington University School of Medicine, St. Louis, MO, United States; ^4^ Division of Endocrinology, Diabetes and Nutrition and Program for Personalized and Genomic Medicine, University of Maryland School of Medicine, Baltimore, MD, United States; ^5^ Duke Center for the Study of Aging and Human Development, Duke University, Durham, NC, United States; ^6^ Durham VA Geriatrics Research Education and Clinical Center, Durham, NC, United States

**Keywords:** aging, Alzheimer’s disease, physiological dysregulation, resilience, axon guidance, synaptic function, genetic interactions, candidate genes

## Abstract

Dysregulation of physiological processes may contribute to Alzheimer’s disease (AD) development. We previously found that an increase in the level of physiological dysregulation (PD) in the aging body is associated with declining resilience and robustness to major diseases. Also, our genome-wide association study found that genes associated with the age-related increase in PD frequently represented pathways implicated in axon guidance and synaptic function, which in turn were linked to AD and related traits (e.g., amyloid, tau, neurodegeneration) in the literature. Here, we tested the hypothesis that genes involved in PD and axon guidance/synapse function may jointly influence onset of AD. We assessed the impact of interactions between SNPs in such genes on AD onset in the Long Life Family Study and sought to replicate the findings in the Health and Retirement Study. We found significant interactions between SNPs in the *UNC5C* and *CNTN6*, and *PLXNA4* and *EPHB2* genes that influenced AD onset in both datasets. Associations with individual SNPs were not statistically significant. Our findings, thus, support a major role of genetic interactions in the heterogeneity of AD and suggest the joint contribution of genes involved in PD and axon guidance/synapse function (essential for the maintenance of complex neural networks) to AD development.

## 1 Introduction

Age is the strongest risk factor for Alzheimer’s disease (AD), suggesting that aging-related changes in the body may contribute to AD development. Indeed, human aging is characterized by an increasing level of dysregulation across body systems, leading to declines in resilience and robustness, with increased susceptibility to multiple health disorders and reduced ability to recover, as people age (Cohen et al., 2013; Arbeev et al., 2016; Whitson et al., 2016; Arbeev et al., 2019; Dansereau et al., 2019; Müller et al., 2019; Chanti-Ketterl et al., 2021; Ukraintseva et al., 2021; Lu et al., 2022). AD has also been linked to the dysregulation of metabolic processes and neural networks (An et al., 2018; de la Monte et al., 2019; Jeong et al., 2019; Yan et al., 2020; Fagiani et al., 2021; Jouini et al., 2021; Nanclares et al., 2021; Joshi et al., 2022; Martínez-Serra et al., 2022), which suggests that the aging-related increase in physiological dysregulation may contribute to the development of AD (Tan et al., 2011; von Bernhardi et al., 2015; Lutshumba et al., 2021; Piehl et al., 2022).

In a number of recent works, we explored a composite index of physiological dysregulation (PD) based on the statistical Mahalanobis distance that measures deviations of multiple biomarkers from their baseline/normal values, thereby reducing the high-dimensional biomarker space into a single PD estimate (Cohen et al., 2014; Arbeev et al., 2016). We found that the rate of increase in PD is a significant predictor of worsening health status manifested by onset of major complex diseases such as cancer, cardiovascular diseases or type 2 diabetes (Arbeev et al., 2019). We also found that genes associated with the increase in PD in our genome-wide association study (GWAS) of the Long Life Family Study participants are significantly enriched for involvement in *axon guidance* and *synaptic function* (Arbeev et al., 2020). Notably, pathways and molecules regulating axon guidance and synaptic function have also been broadly connected to AD and related traits (e.g., amyloid, tau, neurodegeneration) in the literature (Cissé et al., 2011; Jun et al., 2014 Wetzel-Smith et al., 2014; Jiang et al., 2015; Giri et al., 2016; Hashimoto et al., 2016; Kang et al., 2016; Zuko et al., 2016; Oguro-Ando et al., 2017; Han et al., 2018; Li et al., 2018; Liu et al., 2018; Lee et al., 2019; Whelan et al., 2019; Yang et al., 2019; Bamford et al., 2020; Cuestas Torres and Cardenas, 2020; Chen et al., 2021; Zhang et al., 2021). These findings indicate a possibility that genes involved in PD and axon guidance/synaptic function may jointly contribute to onset of AD via intertwined mechanisms. In this paper, we explore whether such genes influence AD onset in older adults, individually or as a result of epistatic interactions, using longitudinal human data from the Long Life Family Study and the Health and Retirement Study.

## 2 Materials and Methods

### 2.1 Data

The Long Life Family Study (LLFS) is a family-based, longitudinal study of healthy aging and longevity that enrolled participants at three United States field centers (Boston, New York, Pittsburgh) and in Denmark. From 2006 to 2009, more than 4,900 participants from 583 families selected for exceptional familial longevity based on the Family Longevity Selection Score (Sebastiani et al., 2009) were enrolled in the study. The second in-person visit of surviving participants from visit 1 and new enrollees was completed in 2014–2017. The third in-person visit started in 2020 and is ongoing (these data are not yet available for this study). At enrollment, the participants provided information on socio-demographic indicators, past and current medical conditions, medication use, and physical and cognitive functioning (Wojczynski et al., 2022). LLFS participants were followed up annually to track their vital and health status. The analyses reported in this paper used the July 2020 release of LLFS data, with the latest recorded follow-up date on 22 July 2019. Ages at the baseline visit were validated using dates of birth from official documents (such as a birth certificate or driver’s license) (Elo et al., 2013) in the United States and through the civil registration system in Denmark. Ages at censoring for those who did not die within the follow-up period were determined from dates of birth and the last follow-up. Information on past and present health conditions (including AD or dementia) was collected during the baseline and follow-up interviews from study participants or proxies (when the participant was unable to provide an answer). The question asked at the baseline visit was, “Please respond “yes” or “no” if you have EVER been told by a doctor that you had this condition.” Similar questions were asked during follow-up interviews (“Please respond “yes” or “no” if you have EVER been told by a doctor that you had this condition since we last interviewed you on … ”). Participants or their proxies were then asked the age at which they were diagnosed or their best guess. Also, during follow-up interviews participants who had cognitive concern were given a dementia questionnaire adopted by the study and were asked if they saw a neurologist and “If “Yes” was the diagnosis Alzheimer’s Disease?” Age at AD or dementia diagnosis (approximating the age of AD onset) was determined by the age they responded affirmatively and, if this information was unavailable, their age when the questionnaire was administered. Ages at censoring, computed as described above, were used in the time-to-event modeling for those who did not report the event during the follow-up.

Written informed consent was obtained from all subjects following protocols approved in the United States by the respective field center’s Institutional Review Boards (IRBs) and, in Denmark, by the Regional Committees on Health Research Ethics for Southern Denmark. In this paper, we performed secondary analyses of LLFS data collected at all field centers. This study was approved by the Duke University Health System IRB.

The University of Michigan’s Health and Retirement Study (HRS) is a longitudinal panel study that surveys a representative sample of approximately 20,000 people in America over the age of 50 every 2 years. The study launched data collection in 1992 and has re-interviewed the original sample of respondents every 2 years. The participants have provided information about income, work, assets, pension plans, health insurance, disability, physical health and functioning, cognitive functioning, and healthcare expenditures. Data were collected from different sources, including in-person, telephone, mail, and the Internet. The target population for the original HRS cohort included all adults in the contiguous United States born during the years 1931–1941 who reside in households, with a 2:1 oversample of African-American and Hispanic populations. The original sample is refreshed with new birth cohorts (51–56 years of age) every 6 years. The sample has been expanded over the years to include a broader range of birth cohorts and described in more detail by Sonnega et al. (2014). The RAND HRS Longitudinal File is an easy-to-use dataset based on the HRS core data. This file was developed at RAND with funding from the National Institute on Aging and the Social Security Administration. This file (version 2016 V1) was used in this study. Entry ages were determined from the first exam date (called “wave” in HRS). Exit ages were determined from ages at death and dates of birth for individuals who died during the follow-up. For individuals who survived the whole study, the lifespan was censored on 31 December 2017 (the latest available date in the sample used). Starting from wave 10 (year 2010), respondents (or proxies, if respondents were not able to provide answers) were asked if they have ever been told they have Alzheimer’s disease or dementia. If the condition was reported at a prior interview, then the question was skipped. Ages of AD onset were determined from the dates of the interview at the respective wave when the condition was first reported and dates of birth. For individuals who did not report the condition, ages at censoring, computed as described above, were used in the time-to-event modeling.

These two datasets are well-suited to serve as discovery and validation sets for two reasons. First, they are complementary because the LLFS is a family-based study in which participants were selected from longevity-enriched families presumably sharing genetic factors likely related to biological aging. Because the HRS is a nationally-representative population-based study, it can be used to determine whether relations detected in LLFS are replicated in the general United States population. Second, the studies use the same genotyping platform (see Section 2.3), thus simplifying the direct validation of discovered associations. As the LLFS sample has predominantly (>99%) White participants, we selected a subsample of White HRS participants for replication (see Section 2.3).

### 2.2 Candidate genes

Table 1 shows the list of candidate genes selected for this study, with the number of SNPs in each gene that remained after the genetic quality control (QC, see Section 2.3). This list includes six genes found in our earlier GWAS (*TRIO*, *ALCAM*, *CADM1*, *CNTN6*, *RTN4*, *PLXNA4*) that were significantly associated with the rate of increase in PD with age and enriched in biological processes of axon guidance/synaptic function (Arbeev et al., 2020). Since AD has been linked to physiological dysregulation and synaptic functioning in other research (Martínez-Serra et al., 2022; Ma et al., 2023; see also *Introduction*), the above genes were selected as candidates for the association analyses with AD risk in this study. We also included in the analysis six additional genes (*PLXNB2*, *EPHA2*, *EPHB2*, *SEMA4D*, *UNC5B*, *UNC5C*) for plexins, semaphorins, ephrins, and receptors to netrins that are major molecular players in axon guidance (during development) and synaptic function (in adults) and have been repeatedly linked to AD and related traits, such as amyloid beta, tau phosphorylation, neurodegeneration, cognitive function and neuroinflammation, in the literature. These additional genes were used as the literature-based candidate AD genes that are involved in axon guidance/synaptic function (Tang et al., 2008; Cissé et al., 2011; Jun et al., 2014; Wetzel-Smith et al., 2014; Jiang et al., 2015; Giri et al., 2016; Hashimoto et al., 2016; Zuko et al., 2016; Han et al., 2018; Li et al., 2018; Liu et al., 2018; McDermott et al., 2018; Lee et al., 2019; Yang et al., 2019; Chen et al., 2021; Zhang et al., 2021; Evans et al., 2022; Ma et al., 2022). The resulting set of twelve candidate genes was, therefore, highly enriched for putative roles in axon guidance, synaptic function, PD, and AD-related traits, and was based on our own recent findings, as well as on the literature (Table 1).

**TABLE 1:** Candidate genes selected for this study.

Gene	Chr	N SNPs (LLFS)	N SNPs (HRS)	Relevant biological processes and health disorders
*ALCAM*	3	103	99	Cell adhesion, migration, axon growth, cancer, immunoglobulins
*CADM1*	11	151	110	Synaptic cell adhesion, axon guidance, cancer prognosis
*CNTN6*	3	494	366	Axon connections, cell adhesion, intellectual disability
*TRIO*	5	199	153	Synaptic function, axon guidance, cognition, cells migration, invasion
*RTN4*	2	111	90	Nerve growth inhibitor, block regeneration
*PLXNA4*	7	391	264	*Plexins* (receptors for semaphorins): axon guidance, Parkinson’s, AD, tau, cancer progression
*PLXNB2*	22	10	9	*Plexins* (receptors for semaphorins): axon guidance, Parkinson’s, AD, tau, cancer progression
*EPHA2*	1	16	15	*Ephrins*: axon guidance, cell migration, cell growth, AD
*EPHB2*	1	140	123	*Ephrins*: axon guidance, cell migration, cell growth, AD
*SEMA4D*	9	116	103	*Semaphorins*: axon guidance, gliosis, AD, cancer progression
*UNC5B*	10	83	66	Receptors for *netrins*: axon guidance, cell migration, apoptosis, neuronal survival, cancer prognosis
*UNC5C*	4	328	256	Receptors for *netrins*: axon guidance, cell migration, apoptosis, AD, cancer prognosis

Notes: Columns: Gene, gene name according to the HUGO Gene Nomenclature Committee (Braschi et al., 2019); Chr, chromosome; N SNPs LLFS, number of genotyped SNPs located in respective gene that were available for analyses in the Long Life Family Study (LLFS); N SNPs HRS, number of genotyped SNPs located in respective gene that were available for analyses in the Health and Retirement Study (HRS). The numbers indicated in the last two columns correspond to SNPs passing the genetic QC procedure (see Section 2.3). “Relevant biological processes and health disorders” are based on the information from PubMed (https://pubmed.ncbi.nlm.nih.gov/) and NCBI Gene (https://www.ncbi.nlm.nih.gov/gene/).

### 2.3 Genotyping, quality control, and selection of SNPs from the data

Blood assays in LLFS were collected via in-person visits and centrally processed at a Laboratory Core (University of Minnesota). Protocols were standardized, monitored, and coordinated through the LLFS Data Management and Coordinating Center. Genotyping in LLFS was performed by the Center for Inherited Disease Research (CIDR) using Illumina Human Omni 2.5 v1 BeadChip array [details on genotyping are provided in Lee et al. (2014)]. Genotyping in HRS was performed by the CIDR using a similar array. Details on genotyping in HRS are provided by Sonnega et al. (2014).

The genetic QC procedure was performed prior to selection of SNPs for analyses based on recommended procedures (Anderson et al., 2010; Marees et al., 2018). For LLFS, the original data contained 5,036 individuals, and among those, 4,693 (2,581 females, 2,112 males) were genotyped with 2,225,478 SNPs available. For HRS, the starting sample was 10,958 (6,285 females, 4,673 males) White participants (as reported in the data) with available genetic data on 2,443,179 SNPs. In both studies, the sample QC check removed individuals with a call rate below 95% and/or heterozygosity rate beyond ± 4 standard deviations (SD) from the mean as well as individuals of divergent ancestry, i.e., those for whom the first two principal components (PC) scores (computed as described below) were beyond 8 SD from the respective mean scores for the HapMap Phase III European reference populations. The SNPs QC check removed duplicated SNPs, variants with missing allele code information, indels, SNPs with a call rate below 95%, minor allele frequency below 1% (in the entire sample and in the sex-specific subsamples), and those with a significant deviation from Hardy-Weinberg equilibrium with *p*-value <10^–10^. In analyses of SNP interactions, an additional criterion was used to keep only SNP pairs that have the sum of the four genotype combinations containing minor alleles to be at least 20 subjects. For LLFS, the resulting sample after QC contained 4,635 individuals of European ancestry (2,548 females, 2,087 males) and 1,468,536 autosomal SNPs, which passed the QC procedure. For HRS, the respective sample was 10,715 participants of European ancestry (6,152 females, 4,563 males) and 1,267,413 post-QC autosomal SNPs. We used these SNPs to select SNPs in the candidate genes shown in Table 1. All available SNPs were first selected if their positions were within the boundary of the genes of interest using information from dbSNP build 151 GRCh38, available from ftp://ftp.ncbi.nlm.nih.gov/snp. SNPs from this initial list were retained for the analyses if they appeared in the post-QC genetic data of the respective dataset. The resulting numbers of SNPs in each candidate gene in LLFS and HRS are shown in Table 1.

The R-package GENESIS version 2.18 (Conomos et al., 2019) was used for the computation of PCs in LLFS using the PC-AiR method (Conomos et al., 2015) to take into account the relatedness among individuals in the LLFS sample. Linkage disequilibrium (LD) pruning was performed before running PC analysis to select a set of independent SNPs for computations of PCs (defined according to the LD threshold 0.2 and a 500 kb sliding window, as implemented in the SNPRelate package (Zheng et al., 2012)). The KING-robust kinship coefficient estimator (Manichaikul et al., 2010) was used to measure ancestry divergence to identify a mutually unrelated and ancestry representative subset of individuals as needed for the PC-AiR algorithm. In HRS, pruning was performed using PLINK (with window size 100 variants, shifted by 10 variants at the end of each step, and a pairwise squared correlation threshold of 0.8 at each step) and then PCs were computed using EIGENSOFT version 6.1.4 (Patterson et al., 2006; Price et al., 2006).

### 2.4 Association analyses of individual SNPs and SNP pairs

We first estimated associations of SNPs in the selected twelve candidate genes (Table 1; Section 2.2) with the onset of AD in LLFS data. Next, we validated associations of the top SNPs identified in LLFS using the HRS data. We estimated the associations of both individual SNPs and epistatic interactions between SNPs in these candidate genes.

LLFS is a family-based study, and as such, it has related individuals. In addition, our time-to-event outcome of interest is the age of AD onset. To account for relatedness between individuals in analyses of time-to-event outcomes, we used a recently developed R-package *coxmeg* (He and Kulminski, 2020) that implements a Cox mixed-effects model for time-to-event outcomes in family-based studies that is substantially faster than existing implementations (e.g., R-package *coxme*). We analyzed individual SNPs in the candidate genes (Table 1), using a block-diagonal family cluster matrix with blocks defined by LLFS family ID. Similarly, we used *coxmeg* to analyze interactions of pairs of SNPs (only pairs of SNPs taken from different candidate genes were analyzed, e.g., one SNP from *ALCAM* and one from *CADM1* but not pairs of SNPs from *ALCAM* alone). For the HRS, we used the standard Cox regression model (R-package *survival*) as the HRS does not contain related individuals.

In addition to genetic variants, the models used in the association analyses included several covariates to adjust for potential confounders. For the LLFS, the following covariates were used: age at first in-person visit (when blood assays were collected), sex (1 - male, 0 - female), country (1 - Denmark, 0 - United States), education (1 - below high school, 0 - otherwise), smoking (smoked >100 cigarettes in a lifetime: yes [1]/no [0]; asked at the baseline visit), and medication use (anti-diabetic, lipid-lowering, anti-hypertensive, angina meds; 1 - used at visit 1 or visit 2, 0 - did not use). For the HRS, we used age at genotyping (which is different from age at first exam in HRS), sex (1 - male, 0 - female), smoking status (1 – ever smoked, 0 – never smoked), and education (1 – below high school, 0 – high school graduate and above). In addition, the top 5 PCs with the largest eigenvalues were included in the regression models to account for the population structure (the number of PCs was determined using scree plots).

Post-analysis QC was performed using *easyQC* (Winkler et al., 2014) to remove erroneous output, such as missing/excessive estimates or standard errors. After the post-analysis QC, we performed an LD-based clumping of results using the PLINK (Price et al., 2006) clumping procedure with an r-squared threshold of 0.1. Because the HRS does not have all covariates that were included in the LLFS analyses, we repeated the LLFS analyses using the shorter list of covariates (age, sex, smoking, education, PCs) from the HRS analyses. The LLFS results were similar to those using the original list of covariates and thus are not included in the paper.

## 3 Results

Characteristics of the LLFS and HRS samples are shown in Table 2. We analyzed 4,273 LLFS participants (2,372 females, 1,901 males). Among these, 176 participants (115 for females, 61 for males) reported a new diagnosis of AD at follow-up. For the HRS, the analytic sample included 10,624 participants (6,104 females, 4,520 males) with 336 new cases of AD during the follow-up period (199 among females, 137 among males). See Notes under Table 2 and Materials and Methods that describe how the analytic sample was constructed from the original sample.

**TABLE 2:** Characteristics of the Long Life Family Study and Health and Retirement Study participants used in the study of associations of SNPs in candidate genes with the onset of Alzheimer’s disease.

Sample characteristics	LLFS	HRS
Number of individuals in the sample	4,273	10,624
Number of AD cases during the follow-up	176	336
Age at baseline (mean ± SD [range])	70.4 ± 15.4 [24, 110]	57.7 ± 8.9 [26, 94]
Age at AD onset (mean ± SD [range])	90.8 ± 8.1 [67, 109]	82.5 ± 7.2 [58, 98]
Follow-up period (mean ± SD [range])	8.8 ± 3.3 [0.17, 13.35]	18.9 ± 6.1 [0.38, 25.73]
Females (%)	55.5	57.5
Participants from US field centers (%)	75.2	–
Low educated participants (below high school) (%)	12.5	4.3
Smokers (%)	42.9	57.2
Medication use: anti-diabetic (%)	7.7	–
Medication use: lipid lowering (%)	37.0	–
Medication use: anti-hypertensive (%)	54.1	–
Medication use: angina meds (%)	32.8	–

Notes: 1) Abbreviations: LLFS, Long Life Family Study; HRS, Health and Retirement Study; AD, Alzheimer’s disease; SD, standard deviation. 2) The table shows the numbers after removal of individuals in the genetic QC procedure it and deletion of individuals with missing values of covariates and outcomes used in the analyses. The initial sample considered for analyses was 5,036 for LLFS, and 10,958 for HRS. 401 (243) individuals were removed in the genetic QC procedure it in LLFS (HRS) (see Section 2.3). Then 362 (91) individuals were removed in LLFS (HRS) because they had at least one missing value of the covariates. Numbers of individuals with missing values of covariates and outcomes in LLFS: AD – 117; age at first exam – 0; sex – 0; country – 0; education – 9; smoking – 15; medication use: anti-diabetic – 243; medication use: lipid lowering – 243; medication use: anti-hypertensive – 243; medication use: angina meds – 243. Numbers of individuals with missing values of covariates and outcomes in HRS: AD ‐ 1; age at first exam – 0; sex – 0; education – 27; smoking – 63.

No SNPs in the candidate genes from Table 1 individually showed a significant association with AD in the LLFS after correction for multiple comparisons (Supplementary Table S1). However, we found statistically significant interactions between SNPs in the candidate genes. Table 3 shows the top results for associations of these interactions with onset of AD in the LLFS (in total, 29,635 SNP pairs remained after post-analysis QC and clumping). Twenty one SNP pairs had a false discovery rate (FDR) less than 0.05 (Table 3; Supplementary Table S2). The *CNTN6*, *UNC5C*, *PLXNA4*, and *EPHB2* genes were broadly involved in the SNPxSNP interactions and appeared in most of the top significant interactions (see Supplementary Figure S1).

**TABLE 3:** SNPxSNP interactions significantly associated with the onset of AD in the LLFS.

SNP1	Chr1	Gene1	EAF1	SNP2	Chr2	Gene2	EAF2	Beta	*p*-value	FDR	N
rs71309807	3	*CNTN6*	0.11	rs9307159	4	*UNC5C*	0.20	1.29	$8.6\times 10^{-7}$	0.024	3,400
rs10273006	7	*PLXNA4*	0.45	rs309502	1	*EPHB2*	0.49	−0.73	$2.3\times 10^{-6}$	0.029	3,402
rs4406027	4	*UNC5C*	0.10	rs73158836	7	*PLXNA4*	0.06	2.14	$3.1\times 10^{-6}$	0.029	3,375
rs62263606	3	*ALCAM*	0.20	rs75167003	9	*SEMA4D*	0.05	2.34	$4.3\times 10^{-6}$	0.030	3,405
rs13149449	4	*UNC5C*	0.07	rs7554093	1	*EPHB2*	0.09	1.42	$5.4\times 10^{-6}$	0.030	3,402
rs58809210	4	*UNC5C*	0.18	rs76756609	3	*CNTN6*	0.06	1.97	$7.8\times 10^{-6}$	0.031	3,403
rs262809	3	*CNTN6*	0.30	rs76347104	11	*CADM1*	0.03	2.10	$7.8\times 10^{-6}$	0.031	3,401
rs3772327	3	*CNTN6*	0.48	rs3772554	3	*ALCAM*	0.17	0.88	$1.0\times 10^{-5}$	0.034	3,400
rs2047888	3	*CNTN6*	0.18	rs7468365	9	*SEMA4D*	0.19	0.95	$1.1\times 10^{-5}$	0.034	3,403
rs57155535	5	*TRIO*	0.07	rs78628725	11	*CADM1*	0.06	1.67	$1.5\times 10^{-5}$	0.041	3,405
rs73818458	3	*CNTN6*	0.13	rs77146700	7	*PLXNA4*	0.04	1.61	$2.1\times 10^{-5}$	0.047	3,393
rs27103	5	*TRIO*	0.26	rs893961	1	*EPHB2*	0.23	0.83	$2.1\times 10^{-5}$	0.047	3,399
rs72746095	5	*TRIO*	0.09	rs79100129	9	*SEMA4D*	0.03	2.59	$2.3\times 10^{-5}$	0.047	3,402
rs17193334	3	*CNTN6*	0.07	rs76347104	11	*CADM1*	0.03	2.72	$2.4\times 10^{-5}$	0.047	3,402
rs10496037	2	*RTN4*	0.113	rs6951616	7	*PLXNA4*	0.449	1.008	$2.7\times 10^{-5}$	0.048	3,389
rs76235219	1	*EPHB2*	0.031	rs79362259	3	*CNTN6*	0.037	3.025	$3.1\times 10^{-5}$	0.048	3,404
rs10891807	11	*CADM1*	0.077	rs4661709	1	*EPHA2*	0.068	2.083	$3.3\times 10^{-5}$	0.048	3,392
rs26081	5	*TRIO*	0.180	rs76235219	1	*EPHB2*	0.031	1.763	$3.6\times 10^{-5}$	0.048	3,399
rs17302526	5	*TRIO*	0.048	rs75167003	9	*SEMA4D*	0.050	3.175	$3.6\times 10^{-5}$	0.048	3,405
rs10999750	10	*UNC5B*	0.086	rs4731850	7	*PLXNA4*	0.229	1.243	$3.6\times 10^{-5}$	0.048	3,403
rs28535173	22	*PLXNB2*	0.260	rs4699850	4	*UNC5C*	0.304	0.692	$3.7\times 10^{-5}$	0.048	3,366

Notes: 1) This table shows results for pairs of SNPs from analyses using the R-package *coxmeg* (see Section 2.4). K (K = 1, 2) in the names of the columns correspond to the respective characteristics of the *K*th SNP, in the pair. 2) Columns: SNPK, single nucleotide polymorphism; ChrK, chromosome; GeneK, gene name according to the HUGO Gene Nomenclature Committee (Braschi et al., 2019); EAFK, effect allele frequency; Beta, regression coefficient for the interaction between SNP1 and SNP2; *p*-value, unadjusted *p*-value for the interaction (corresponding to the null hypothesis that the regression coefficient for the interaction is zero); FDR, false discovery rate (Benjamini and Hochberg, 1995) computed from *p*-values for the interaction; N, number of individuals in the analyzed sample. The table shows only the results with FDR<0.05. Note that regression coefficients and *p*-values for the main terms for SNP1 and SNP2 along with some additional information can be found in Supplementary Table S2.

Table 4 shows the results of genetic interactions in the HRS data that support (with *p*-values <0.05) the top two SNP pairs in the *CNTN6* – *UNC5C* and *PLXNA4* – *EPHB2* pairs found in the LLFS (Table 3). These SNPs are in LD with the top SNPs found in the LLFS (see Notes under Table 4). Note that the interactions between the *CNTN6* – *UNC5C* and *PLXNA4* – *EPHB2* genes in the HRS also include other SNPs that are not in LD with the SNPs in the genes found in the LLFS (Supplementary Figure S1). This indicates a broad involvement of interactions between these genes in AD, which is not limited by the few specific SNPs. Indeed, different SNPs in the same gene may sometimes similarly influence its expression or protein yield and thus they can be potentially interchangeable in terms of their biological effects (Yashin et al., 2012). Such interactions should also be explored in future studies at the gene level aggregating many SNPs to better understand how multiple functionally similar SNPs may contribute to gene-by-gene interactions that influence AD onset.

**TABLE 4:** HRS results supporting the top significant SNP pairs in CNTN6 and UNC5C, and PLXNA4 and EPHB2 genes found in LLFS.

SNP1(LLFS)	GENE1	SNP2 (LLFS)	GENE2	SNP1(HRS)	SNP2(HRS)	*R* ^2^ SNP1	D^’^ SNP1	*R* ^2^ SNP2	D^’^ SNP2	P(HRS) SNP1xSNP2
rs71309807	*CNTN6*	rs9307159	*UNC5C*	rs13097143	rs1872129	0.22	0.90	0.25	0.95	0.0286
rs71309807	*CNTN6*	rs9307159	*UNC5C*	rs12107538	rs17023470	0.14	0.91	0.14	0.50	0.0297
rs71309807	*CNTN6*	rs9307159	*UNC5C*	rs10510165	rs1872129	0.23	0.91	0.25	0.95	0.0307
rs71309807	*CNTN6*	rs9307159	*UNC5C*	rs3772351	rs1872129	0.17	0.92	0.25	0.95	0.0457
rs71309807	*CNTN6*	rs9307159	*UNC5C*	rs11128578	rs1872129	0.17	0.92	0.25	0.95	0.0480
rs10273006	*PLXNA4*	rs309502	*EPHB2*	rs10229073	rs309495	0.13	0.99	0.50	0.97	0.0051
rs10273006	*PLXNA4*	rs309502	*EPHB2*	rs10229073	rs309543	0.13	0.99	0.45	0.87	0.0173
rs10273006	*PLXNA4*	rs309502	*EPHB2*	rs6957896	rs309495	0.15	0.52	0.50	0.97	0.0303
rs10273006	*PLXNA4*	rs309502	*EPHB2*	rs1499300	rs309495	0.15	0.88	0.50	0.97	0.0346

Notes: 1) This table shows results of replication of our top two findings from LLFS, shown in Table 3. 2) Columns SNP1 (LLFS), GENE1, SNP2 (LLFS), and GENE2 contain rs numbers and gene names for respective pairs of SNPs, identified in LLFS. Columns SNP1 (HRS) and SNP2 (HRS) show SNPs, from respective genes identified in HRS data, and columns *R* ^2^ SNP1, D′ SNP1, *R* ^2^ SNP2, and D’ SNP2 display respective linkage disequilibrium measures between those SNPs and the SNPs shown in columns SNP1 (LLFS) and SNP2 (LLFS). Column P (HRS) SNP1 x SNP2 presents *p*-values for the null hypotheses of zero interaction term for the SNPs in columns SNP1 (HRS) and SNP2 (HRS).

## 4 Discussion

We estimated associations of individual SNPs in candidate genes involved in PD and axon guidance, as well as SNPxSNP interactions, with AD onset among LLFS and HRS participants. Our analysis revealed significant interactions between the SNPs in the *UNC5C*, *CNTN6*, *PLXNA4,* and *EPHB2* genes that influenced AD onset in both datasets. For the LLFS, the top significant interaction was observed between rs9307159 (*UNC5C*) and rs71309807 (*CNTN6*) (HR = 3.62; Bonferroni *p*-value: 0.026). The same gene pair was also among the top significant interactions associated with AD in the HRS. Results for individual SNPs were not significant. Our findings, thus, strongly support a major role of genetic interactions in the heterogeneity of AD, including the interactions among genes involved in aging-related increase in physiological dysregulation and changes in axon guidance/synaptic functioning.

Our results also show that selecting candidate genes based on their involvement in the same biological process relevant to AD is a viable strategy for identifying significant genetic interactions associated with AD. Indeed, molecular products of the *UNC5C*, *PLXNA4*, and *EPHB2* genes are jointly involved in axon guidance (in development) and synaptic plasticity (in adults), essential for the maintenance of complex neural networks. These genes and their products have also been linked to AD and related traits in the literature (Cissé et al., 2011; Barthet et al., 2013; Jun et al., 2014 Wetzel-Smith et al., 2014; Giri et al., 2016; Hashimoto et al., 2016; Kang et al., 2016; Sun et al., 2016; van Dijken et al., 2017; Han et al., 2018; Li et al., 2018; Liu et al., 2018; Lee et al., 2019; Yang et al., 2019; Chen et al., 2021). Results of our analyses suggest that such genes are plausible candidates for exploring the role of genetic interactions in AD heterogeneity. One hypothetical mechanism connecting AD with the axon guidance genes could be reactivation in the aging body of the developmental synaptic pruning program involving such genes, which may lead to synapse loss and subsequent neurodegeneration (Vanderhaeghen and Cheng, 2010). As for *CNTN6*, it is a member of the contactins family of neuronal membrane proteins that function as cell adhesion molecules playing roles in the formation of axon connections, neurite outgrowth, synaptogenesis, cell survival, and neural circuit formation and regeneration (Oguro-Ando et al., 2017; Chatterjee et al., 2019). Various studies have implicated *CNTN6*, and more broadly contactins as a group, in metabolic, neurological, and mental health conditions, brain disorders, intellectual disability, depression, and AD-related traits (Jia et al., 2017; Oguro-Ando et al., 2017; Chatterjee et al., 2019; Bamford et al., 2020; Morris et al., 2020). All genes selected for the interaction analysis in this study are involved in the same biological processes relevant to AD, likely contributing to their propensity for interactions affecting AD risk.

Earlier we emphasized that the increase in physiological dysregulation is a fundamental feature of human aging contributing to declines in both resistance to disease occurrence (robustness), and ability to recover and survive after disease occurs (resilience) (Arbeev et al., 2019; Ukraintseva et al., 2021). If so, then the PD-related genes, and their interactions, may potentially influence not only AD onset, but also survival after it, as well as chances of extreme longevity. Therefore, a logical next step would be to investigate associations of the PD-related genes with survival outcomes at different age intervals. Given that the age-related increase in PD may affect the functioning of multiple organs, the PD-related genetic variants might also be associated with comorbidities, offering another avenue in this line of research.

We acknowledge that this study has some limitations. First, our outcome variable is an approximated age of onset derived from questionnaires. As these data are recorded during the interviews, they may not accurately reflect true ages of onset. This is a common limitation of the studies using such outcomes. Second, our studied samples were White, so that more analyses (and data) are necessary to investigate if the results hold for other populations. Third, our samples have relatively small numbers of AD cases. Therefore, additional validation studies are needed to determine if the interactions among *UNC5C*, *CNTN6*, *PLXNA4*, and *EPHB2* genes can influence AD onset in other, larger-scale, datasets with time-to-event information on ages of AD onset.

In conclusion, interactions between SNPs (but not individual SNPs) in genes involved in increase in physiological dysregulation with age, a crucial feature of biological aging, and in axon guidance/synaptic functioning, were significantly associated with the age of AD onset in this study. Our findings support the idea that genetic interactions contribute to the heterogeneity of late-onset Alzheimer’s disease. Inherent differences in the rate of PD change with age appear to interact with one’s genetic propensity for synaptic dysfunction in determining AD risk. The results of our study also indicate that system-level changes in the aging body, such as the whole-body increase in physiological dysregulation, might contribute to the brain changes required for AD development.

## Data Availability

Publicly available datasets were analyzed in this study. This data can be found here: the database of Genotypes and Phenotypes (dbGaP) provides access to phenotypic and genetic LLFS data (dbGaP Study Accession: phs000397.v3.p3). The HRS data were provided by dbGaP (dbGaP Study Accession: phs000428.v2.p2) and the University of Michigan (https://hrs.isr.umich.edu/).
