# Supplementary material for: Interactions between genes involved in physiological dysregulation and axon guidance: role in Alzheimer’s disease
Source: Front Genet. 2023 Aug 31;14:1236509. doi: 10.3389/fgene.2023.1236509 (PMC10500346; doi:10.3389/fgene.2023.1236509)
Supplement: Supplementary file 1 [file DataSheet1.docx]

Supplementary Material

# Supplementary Figures and Tables

## Supplementary Figures

| **a**  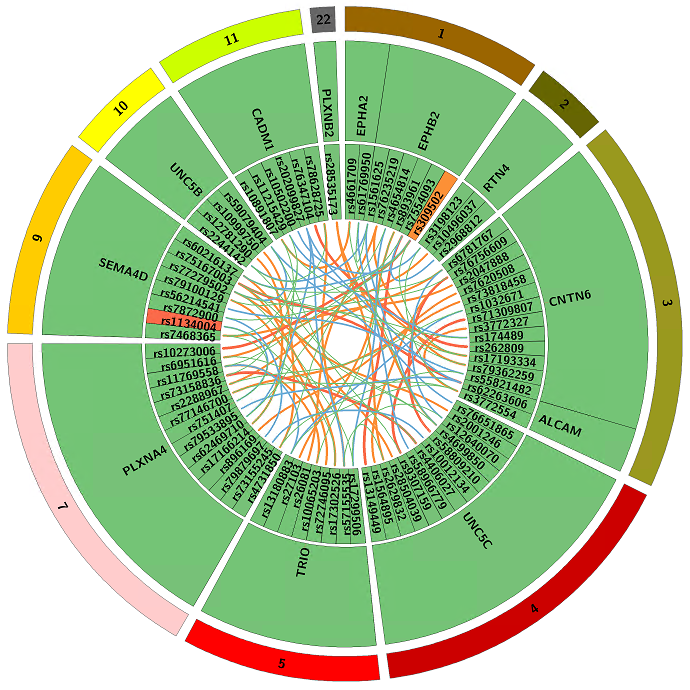 | **b**  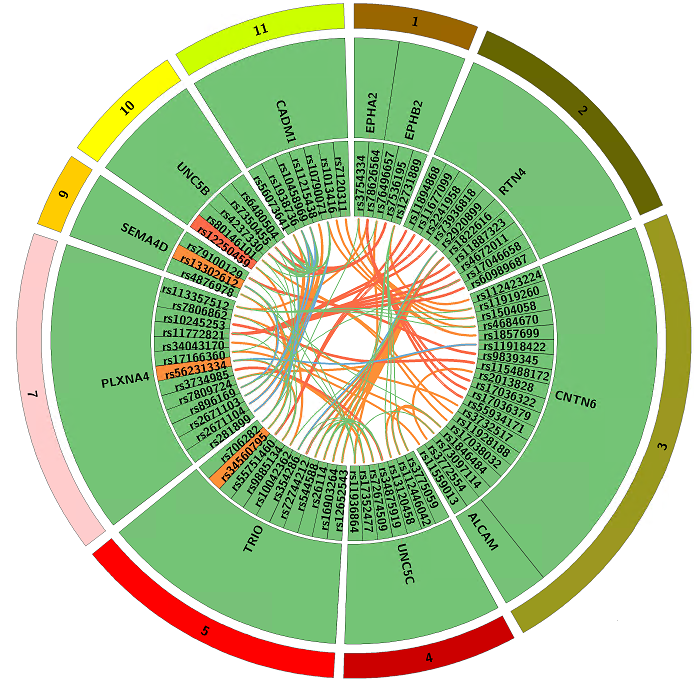 |
| --- | --- |

**Supplementary Figure 1.** Circos plots showing the top interacting SNP pairs that were associated with the onset of Alzheimer’s disease in the Long Life Family Study (**a**, left side) and the Health and Retirement Study (**b**, right side) data. In this analysis, we used all genotyped SNPs in candidate genes described in the main text. Interaction line *p*-values: **red** < 10^-5^, 10^-5^ < **orange** < 10^-4^, 10^-4^ < **blue** < 10^-3^, **green** > 10^-3^. Single SNP *p*-values: **red** < 10^-5^, 10^-5^ < **orange** < 10^-4^, 10^-4^ < **blue** < 0.05, **green** >0.05.

## Supplementary Tables

**Supplementary Table 1.** Top ranked SNPs from results of the analysis of associations with the onset of Alzheimer’s disease (AD) in the Long Life Family Study. This analysis used individual SNPs in candidate genes from Table 1.

| **SNP** | **Chr** | **Gene** | **EA/OA** | **EAF** | **Beta** | **SE** | **P-value** | **FDR** | **N** |
| --- | --- | --- | --- | --- | --- | --- | --- | --- | --- |
| rs62229343 | 3 | *CNTN6* | C/T | 0.051 | 0.692 | 0.209 | $9.4\times{10}^{-4}$ | 0.982 | 3400 |
| rs1134004 | 9 | *SEMA4D* | T/C | 0.233 | 0.356 | 0.117 | 0.0023 | 0.982 | 3405 |
| rs2886164 | 3 | *CNTN6* | T/C | 0.357 | -0.343 | 0.116 | 0.0031 | 0.982 | 3404 |
| rs77205680 | 7 | *PLXNA4* | G/A | 0.034 | 0.642 | 0.226 | 0.0045 | 0.982 | 3397 |
| rs2047888 | 3 | *CNTN6* | T/C | 0.181 | 0.331 | 0.123 | 0.0071 | 0.982 | 3403 |
| rs114502775 | 3 | *CNTN6* | A/G | 0.017 | 0.791 | 0.296 | 0.0075 | 0.982 | 3404 |
| rs115943443 | 7 | *PLXNA4* | A/C | 0.035 | 0.619 | 0.244 | 0.011 | 0.982 | 3394 |

**Notes: SNP** – single nucleotide polymorphism; **Chr** – chromosome; **Gene** – gene name according to the HUGO Gene Nomenclature Committee (Braschi et al., 2019); **EA/OA** – effect/other allele (Wootton and Sallis, 2020); **EAF** – effect allele frequency; **Beta** – regression coefficient for SNP; **SE** – standard error of Beta; **P-value** – unadjusted *p*-value (corresponding to the null hypothesis that the SNP regression coefficient is zero); **FDR** – false discovery rate (Benjamini and Hochberg, 1995) computed from *p*-values for the interaction; **N** – number of individuals in the analyzed sample. Only the variants with smallest *p*-value from groups of SNPs in high LD in Whites (based on EUR - 5 European populations in LDlink, LDpair Tool, <https://ldlink.nci.nih.gov/?tab=ldpair>) are shown in the table.

**Supplementary Table 2.** Detailed information on the top ranked SNP pairs from the interaction association results between SNPs in the selected candidate genes (Table 1) with the onset of AD in the LLFS.

| **SNP** | **Chr** | **Gene** | **EA/OA** | **EAF** | **Beta Indiv.** | **SE Indiv.** | **P Indiv.** | **Beta Inter.** | **SE Inter.** | **P Inter.** | **FDR Inter.** | **N** |
| --- | --- | --- | --- | --- | --- | --- | --- | --- | --- | --- | --- | --- |
| rs71309807 | 3 | *CNTN6* | G/A | 0.112 | -0.619 | 0.242 | $1.0\times{10}^{-2}$ | 1.287 | 0.262 | $8.6\times{10}^{-7}$ | 0.0237 | 3400 |
| rs9307159 | 4 | *UNC5C* | T/C | 0.202 | -0.378 | 0.172 | $2.8\times{10}^{-2}$ |  |  |  |  |  |
|  |  |  |  |  |  |  |  |  |  |  |  |  |
| rs10273006 | 7 | *PLXNA4* | G/A | 0.452 | 0.575 | 0.180 | $1.4\times{10}^{-3}$ | -0.734 | 0.155 | $2.3\times{10}^{-6}$ | 0.0284 | 3402 |
| rs309502 | 1 | *EPHB2* | C/T | 0.488 | 0.671 | 0.172 | $9.5\times{10}^{-5}$ |  |  |  |  |  |
|  |  |  |  |  |  |  |  |  |  |  |  |  |
| rs4406027 | 4 | *UNC5C* | C/T | 0.103 | -0.239 | 0.221 | $2.8\times{10}^{-1}$ | 2.144 | 0.460 | $3.1\times{10}^{-6}$ | 0.0284 | 3375 |
| rs73158836 | 7 | *PLXNA4* | T/C | 0.056 | -0.671 | 0.313 | $3.2\times{10}^{-2}$ |  |  |  |  |  |
|  |  |  |  |  |  |  |  |  |  |  |  |  |
| rs62263606 | 3 | *ALCAM* | T/C | 0.201 | -0.103 | 0.138 | $4.6\times{10}^{-1}$ | 2.341 | 0.509 | $4.3\times{10}^{-6}$ | 0.0295 | 3405 |
| rs75167003 | 9 | *SEMA4D* | C/A | 0.050 | -1.867 | 0.594 | $1.7\times{10}^{-3}$ |  |  |  |  |  |
|  |  |  |  |  |  |  |  |  |  |  |  |  |
| rs13149449 | 4 | *UNC5C* | A/G | 0.065 | -0.684 | 0.293 | $2.0\times{10}^{-2}$ | 1.418 | 0.312 | $5.4\times{10}^{-6}$ | 0.0298 | 3402 |
| rs7554093 | 1 | *EPHB2* | G/A | 0.093 | -0.400 | 0.218 | $6.7\times{10}^{-2}$ |  |  |  |  |  |
|  |  |  |  |  |  |  |  |  |  |  |  |  |
| rs58809210 | 4 | *UNC5C* | A/G | 0.181 | -0.207 | 0.156 | $1.8\times{10}^{-1}$ | 1.971 | 0.441 | $7.8\times{10}^{-6}$ | 0.0307 | 3403 |
| rs76756609 | 3 | *CNTN6* | A/G | 0.055 | -1.452 | 0.467 | $1.9\times{10}^{-3}$ |  |  |  |  |  |
|  |  |  |  |  |  |  |  |  |  |  |  |  |
| rs262809 | 3 | *CNTN6* | T/C | 0.304 | -0.289 | 0.131 | $2.7\times{10}^{-2}$ | 2.095 | 0.469 | $7.8\times{10}^{-6}$ | 0.0307 | 3401 |
| rs76347104 | 11 | *CADM1* | T/C | 0.029 | -1.870 | 0.802 | $2.0\times{10}^{-2}$ |  |  |  |  |  |
|  |  |  |  |  |  |  |  |  |  |  |  |  |
| rs3772327 | 3 | *CNTN6* | C/T | 0.475 | -0.273 | 0.128 | $3.2\times{10}^{-2}$ | 0.879 | 0.199 | $1.0\times{10}^{-5}$ | 0.0341 | 3400 |
| rs3772554 | 3 | *ALCAM* | T/C | 0.173 | -0.927 | 0.285 | $1.2\times{10}^{-3}$ |  |  |  |  |  |
|  |  |  |  |  |  |  |  |  |  |  |  |  |
| rs2047888 | 3 | *CNTN6* | T/C | 0.181 | -0.005 | 0.154 | $9.8\times{10}^{-1}$ | 0.951 | 0.216 | $1.1\times{10}^{-5}$ | 0.0341 | 3403 |
| rs7468365 | 9 | *SEMA4D* | G/A | 0.189 | -0.441 | 0.200 | $2.8\times{10}^{-2}$ |  |  |  |  |  |
|  |  |  |  |  |  |  |  |  |  |  |  |  |
|  |  |  |  |  |  |  |  |  |  |  |  |  |
| rs57155535 | 5 | *TRIO* | C/T | 0.067 | 0.139 | 0.205 | $5.0\times{10}^{-1}$ | 1.669 | 0.386 | $1.5\times{10}^{-5}$ | 0.0414 | 3405 |
| rs78628725 | 11 | *CADM1* | C/T | 0.060 | -0.287 | 0.265 | $2.8\times{10}^{-1}$ |  |  |  |  |  |
|  |  |  |  |  |  |  |  |  |  |  |  |  |
| rs73818458 | 3 | *CNTN6* | G/T | 0.125 | -0.169 | 0.175 | $3.3\times{10}^{-1}$ | 1.611 | 0.379 | $2.1\times{10}^{-5}$ | 0.0468 | 3393 |
| rs77146700 | 7 | *PLXNA4* | A/G | 0.037 | -0.558 | 0.349 | $1.1\times{10}^{-1}$ |  |  |  |  |  |
|  |  |  |  |  |  |  |  |  |  |  |  |  |
| rs27103 | 5 | *TRIO* | C/T | 0.262 | -0.372 | 0.169 | $2.8\times{10}^{-2}$ | 0.826 | 0.194 | $2.1\times{10}^{-5}$ | 0.0468 | 3399 |
| rs893961 | 1 | *EPHB2* | G/A | 0.229 | -0.416 | 0.180 | $2.1\times{10}^{-2}$ |  |  |  |  |  |
|  |  |  |  |  |  |  |  |  |  |  |  |  |
| rs72746095 | 5 | *TRIO* | A/G | 0.086 | -0.135 | 0.197 | $4.9\times{10}^{-1}$ | 2.585 | 0.611 | $2.3\times{10}^{-5}$ | 0.0468 | 3402 |
| rs79100129 | 9 | *SEMA4D* | T/C | 0.026 | -1.451 | 0.729 | $4.7\times{10}^{-2}$ |  |  |  |  |  |
|  |  |  |  |  |  |  |  |  |  |  |  |  |
| rs17193334 | 3 | *CNTN6* | A/G | 0.068 | -0.151 | 0.249 | $5.4\times{10}^{-1}$ | 2.717 | 0.643 | $2.4\times{10}^{-5}$ | 0.0468 | 3402 |
| rs76347104 | 11 | *CADM1* | T/C | 0.029 | -0.198 | 0.415 | $6.3\times{10}^{-1}$ |  |  |  |  |  |
|  |  |  |  |  |  |  |  |  |  |  |  |  |
| rs10496037 | 2 | *RTN4* | T/C | 0.113 | -1.189 | 0.348 | $6.4\times{10}^{-4}$ | 1.008 | 0.240 | $2.7\times{10}^{-5}$ | 0.0484 | 3389 |
| rs6951616 | 7 | *PLXNA4* | C/T | 0.449 | -0.357 | 0.121 | $3.2\times{10}^{-3}$ |  |  |  |  |  |
|  |  |  |  |  |  |  |  |  |  |  |  |  |
| rs76235219 | 1 | *EPHB2* | T/C | 0.031 | -0.451 | 0.365 | $2.2\times{10}^{-1}$ | 3.025 | 0.726 | $3.1\times{10}^{-5}$ | 0.0484 | 3404 |
| rs79362259 | 3 | *CNTN6* | G/A | 0.037 | -0.665 | 0.374 | $7.6\times{10}^{-2}$ |  |  |  |  |  |
|  |  |  |  |  |  |  |  |  |  |  |  |  |
| rs10891807 | 11 | *CADM1* | C/T | 0.077 | -0.200 | 0.216 | $3.5\times{10}^{-1}$ | 2.083 | 0.502 | $3.3\times{10}^{-5}$ | 0.0484 | 3392 |
| rs4661709 | 1 | *EPHA2* | T/C | 0.068 | -0.620 | 0.321 | $5.3\times{10}^{-2}$ |  |  |  |  |  |
|  |  |  |  |  |  |  |  |  |  |  |  |  |
| rs26081 | 5 | *TRIO* | T/A | 0.180 | -0.128 | 0.152 | $4.0\times{10}^{-1}$ | 1.763 | 0.427 | $3.6\times{10}^{-5}$ | 0.0484 | 3399 |
| rs76235219 | 1 | *EPHB2* | T/C | 0.031 | -0.851 | 0.448 | $5.8\times{10}^{-2}$ |  |  |  |  |  |
|  |  |  |  |  |  |  |  |  |  |  |  |  |
| rs17302526 | 5 | *TRIO* | T/C | 0.048 | -0.647 | 0.387 | $9.5\times{10}^{-2}$ | 3.175 | 0.768 | $3.6\times{10}^{-5}$ | 0.0484 | 3405 |
| rs75167003 | 9 | *SEMA4D* | C/A | 0.050 | -0.504 | 0.324 | $1.2\times{10}^{-1}$ |  |  |  |  |  |
|  |  |  |  |  |  |  |  |  |  |  |  |  |
| rs10999750 | 10 | *UNC5B* | T/G | 0.086 | -0.932 | 0.363 | $1.0\times{10}^{-2}$ | 1.243 | 0.301 | $3.6\times{10}^{-5}$ | 0.0484 | 3403 |
| rs4731850 | 7 | *PLXNA4* | T/C | 0.229 | -0.045 | 0.136 | $7.4\times{10}^{-1}$ |  |  |  |  |  |
|  |  |  |  |  |  |  |  |  |  |  |  |  |
|  |  |  |  |  |  |  |  |  |  |  |  |  |
| rs28535173 | 22 | *PLXNB2* | G/T | 0.260 | -0.580 | 0.185 | $1.8\times{10}^{-3}$ | 0.692 | 0.168 | $3.7\times{10}^{-5}$ | 0.0484 | 3366 |
| rs4699850 | 4 | *UNC5C* | C/A | 0.304 | -0.263 | 0.158 | $9.5\times{10}^{-2}$ |  |  |  |  |  |

**Notes:** 1) The table shows top results of estimates for pairs of SNPs (separated by empty lines for better visibility) from analyses using *coxmeg* (see Section 2.4). Respective rows contain information for each SNP from the pair. Information pertaining to the pair (such as *p*-value for the interaction) is shown in the middle of respective lines. 2) Columns: **SNP** – single nucleotide polymorphism; **Chr** – chromosome; **Gene** – gene name according to the HUGO Gene Nomenclature Committee (Braschi et al., 2019); **EA/OA** – effect/other allele (Wootton and Sallis, 2020); **EAF** – effect allele frequency; **Beta** **Indiv.** – regression coefficient for individual SNPs; **SE Indiv.** – standard error of Beta Indiv.; **P Indiv.** – unadjusted *p*-value for individual SNPs (corresponding to the null hypothesis that the regression coefficient for the SNP is zero); **Beta** **Inter.** – regression coefficient for the interaction between two SNPs in the pair; **SE Inter.** – standard error of Beta Inter.; **P Inter.** – unadjusted *p*-value for the interaction (corresponding to the null hypothesis that the regression coefficient for the interaction is zero); **FDR Inter.** – false discovery rate (FDR) (Benjamini and Hochberg, 1995) computed from *p*-values for the interaction; **N** – number of individuals in the analyzed sample. The table shows all pairs with FDR<0.05.

**References:**

Benjamini, Y., and Hochberg, Y. (1995). Controlling the false discovery rate: a practical and powerful approach to multiple testing. *Journal of the Royal Statistical Society: Series B* 57**,** 289-300.

Braschi, B., Denny, P., Gray, K., Jones, T., Seal, R., Tweedie, S., Yates, B., and Bruford, E. (2019). Genenames.org: the HGNC and VGNC resources in 2019. *Nucleic Acids Res* 47**,** D786-d792.

Wootton, R.E., and Sallis, H.M. (2020). Let’s call it the effect allele: a suggestion for GWAS naming conventions. *International Journal of Epidemiology* 49**,** 1734-1735.
